# The Operation of Arytenectomy Assisted by the Electrical Lamp

**Published:** 1897-06

**Authors:** W. H. Arrowsmith

**Affiliations:** Jersey City, N. J.


### THE OPERATION OF ARYTENECTOMY ASSISTED BY THE ELECTRICAL LAMP.

By W. H. Arrowsmith, D.V.S.,
JERSEY CITY, N. J.

The operation of arytenectomy has always been considered difficult and unsatisfactory, because the cutting out of the arytenoid cartilages must be performed at a distance of from four to six inches from the opening in the trachea, the opening itself being almost filled up with the operator's hands, speculum, and instruments.

Being unable to see, the knife may slip or curved-scissors' points penetrate the soft parts, or the tenaculums or forceps grasp portions of the mucous membranes with the cartilages, and the tissues unfortunately injured which one wishes to preserve. When the cartilages are enucleated the effort to take up the mucous membrane properly with catgut suture becomes almost impossible, on account of the inability to see because of the small aperture and the frequent accumulation of blood-clots; and when the patient is let up, and the parts are healed, to your surprise and regret, the breathing is still imperfect.

During the latter part of December, 1896, the writer received at his hospital a valuable mare, five years old, to be operated on for ossification of the arytenoid cartilages, and determined to improve on the usual methods of performing arytenectomy, and succeeded after several attempts in having a practical electrician make an electric lamp, the globe of which was only one-fourth of an inch in diameter and affixed to the end of a hard black-rubber handle twelve inches long, through which passed two fine copper wires connecting with the platinum loop in the globe. At the other end of the handle was a thumb-press so placed that the light might be turned on and off at the will of the operator. The wires were then connected to three dry batteries, and we were prepared to illuminate the laryngeal cavity.

The mare was prepared, thrown, and placed upon her back; a tracheotomy-tube inserted midway down the trachea; the trachea was then opened just below the cricoid cartilage, by separating the muscles and cutting six rings of it, and the parts held open by two spoon-shaped blunt tenaculums, especially made for that purpose. The trachea between the upper opening and the tracheotomy-tube was filled with soft sterilized sponges, to which cords were attached and fastened outside, so that the sponges could not slip down into the cavity of the lung. The lamp was then inserted into the laryngeal cavity and the light turned on, when all the parts were distinctly seen, and each cut of the knife or scissors could be followed, and the forceps placed exactly where the operator wished; the whole ossified cartilages were enucleated and the mucous membrane taken up with catgut sutures and properly tied, each cartilaginous ring drawn together with catgut sutures and the muscles with silver sutures. The only interference with the operation was at times the accumulation of blood in the laryngeal cavity and on the globe of the lamp. The mare made a rapid and complete recovery, and three weeks later she could be speeded without any noise or emphatic breathing, and was then turned out.

